# Neuromelanin in Parkinson’s Disease: Tyrosine Hydroxylase and Tyrosinase

**DOI:** 10.3390/ijms23084176

**Published:** 2022-04-10

**Authors:** Toshiharu Nagatsu, Akira Nakashima, Hirohisa Watanabe, Shosuke Ito, Kazumasa Wakamatsu

**Affiliations:** 1 Center for Research Promotion and Support, School of Medicine, Fujita Health University, Toyoake 470-1192, Aichi, Japan; 2 Department of Physiological Chemistry, School of Medicine, Fujita Health University, Toyoake 470-1192, Aichi, Japan; anakashi@fujita-hu.ac.jp; 3 Department of Neurology, School of Medicine, Fujita Health University, Toyoake 470-1192, Aichi, Japan; hirohisa.watanabe@fujita-hu.ac.jp; 4 Institute for Melanin Chemistry, Fujita Health University, Toyoake 470-1192, Aichi, Japan; sito@fujita-hu.ac.jp (S.I.); kwaka@fujita-hu.ac.jp (K.W.)

**Keywords:** dopamine, locus coeruleus, melanin, neuromelanin, norepinephrine, Parkinson’s disease, substantia nigra, tyrosinase, tyrosine hydroxylase

## Abstract

Parkinson’s disease (PD) is an aging-related disease and the second most common neurodegenerative disease after Alzheimer’s disease. The main symptoms of PD are movement disorders accompanied with deficiency of neurotransmitter dopamine (DA) in the striatum due to cell death of the nigrostriatal DA neurons. Two main histopathological hallmarks exist in PD: cytosolic inclusion bodies termed Lewy bodies that mainly consist of α-synuclein protein, the oligomers of which produced by misfolding are regarded to be neurotoxic, causing DA cell death; and black pigments termed neuromelanin (NM) that are contained in DA neurons and markedly decrease in PD. The synthesis of human NM is regarded to be similar to that of melanin in melanocytes; melanin synthesis in skin is via DOPAquinone (DQ) by tyrosinase, whereas NM synthesis in DA neurons is via DAquinone (DAQ) by tyrosine hydroxylase (TH) and aromatic L-amino acid decarboxylase (AADC). DA in cytoplasm is highly reactive and is assumed to be oxidized spontaneously or by an unidentified tyrosinase to DAQ and then, synthesized to NM. Intracellular NM accumulation above a specific threshold has been reported to be associated with DA neuron death and PD phenotypes. This review reports recent progress in the biosynthesis and pathophysiology of NM in PD.

## 1. Neuromelanin (NM) in Parkinson’s Disease

Parkinson’s disease (PD) is a human-specific, progressive, aging-related disease, and the second most common neurodegenerative disease after Alzheimer’s disease [1]. In 1817, James Parkinson in London published “An Essay on the Shaking Palsy”, the first comprehensive clinical description of a disorder later named Parkinson’s disease. The main symptoms of PD are motor symptoms, such as tremor, bradykinesia, rigidity, and postural instability, as well as non-motor symptoms including anosmia, constipation, insomnia, REM-sleep behavioral disorders (RBD), anxiety, depression, fatigue, and cognitive impairment [1]. Most PD is sporadic without a familial history (sPD). Only 5–15 percent of cases are familial PD (fPD) [2,3]. The pathophysiology of PD was investigated by biochemical analysis of post-mortem PD brains during the middle of 20th century [4,5,6,7]. Although the pathophysiology of PD still remains unknown, sPD is thought to be caused by combined effects of environmental and genetic factors. The main symptoms of PD, which is a movement disorder, are known to be caused by a decrease in neurotransmitter dopamine (DA) in the striatum in the basal ganglia due to neurodegeneration of nigrostriatal DA neurons, and supplementation of DA by the direct precursor L-3,4-dihydroxyphenylalanine (L-DOPA) is still the gold standard of pharmacotherapy of PD after five decades since 1970s [1,7,8]. L-DOPA treatment is highly effective for alleviating many core symptoms of PD, but it does not prevent the progression of neurodegeneration and later results in a decrease in efficacy and various side effects such as dyskinesia [7,8].

The discovery of the causative or susceptibility genes of various fPD, since the end of 20th century, has greatly promoted the elucidation of molecular mechanism of sPD [3]; fPD is termed in the order of discovery of the gene loci such as PARK1 (α-synuclein, *SNCA* [9,10]) and PARK2 (parkin, *PRKN* [3,11,12]). More than 20 PARKs have been reported. The abbreviation PARK is derived from the name PARKinson. Mutations in some genes in fPD are considered to be causative and also related to susceptibility loci in sPD, for example, α-synuclein gene (*SNCA* and *PARK1*) [9,10], parkin (*PARK2*) [3,11], PTEN-induced putative kinase 1 (*PINK1* and *PARK6*) [13,14], and leucine-rich repeat kinase 2 (*LRRK2* and *PARK8*) [15,16,17,18].

**Figure 1:**
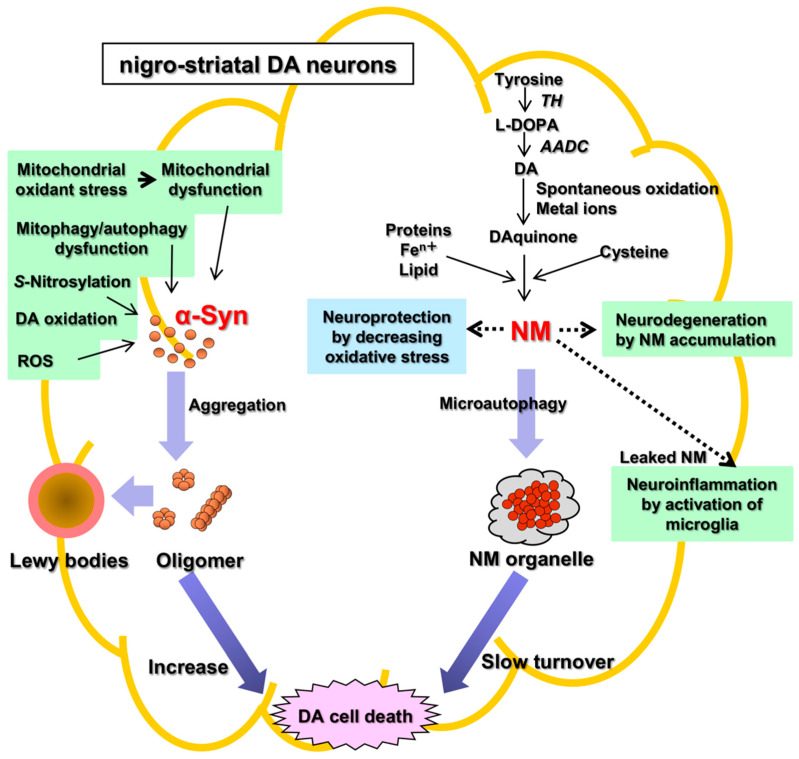
Two histopathological hallmarks in PD in the nigrostriatal DA. Fibrillar oligomers of α-Syn produced by misfolding are presumed to be neurotoxic and to cause DA cell death. Neuromelanin (NM) is also related to neurodegeneration and DA cell death, because NM attenuates the oxidative stress for neuroprotection. α-Syn, α-synuclein; NM, neuromelanin; TH, tyrosine hydroxylase; AADC, aromatic amino acid decarboxylase; ROS, reactive oxygen species.

There are two main histopathological hallmarks in PD in the degenerating nigrostriatal DA neurons, i.e., Lewy bodies and reduction of neuromelanin (NM) in substantia nigra (SN) (Figure 1): (1) Friedrich Heinrich described cytosolic inclusion bodies termed Lewy bodies in 1912 [19]. Lewy bodies contain α-synuclein protein as the main protein component, and the fibrillar oligomers of α-synuclein protein produced by misfolding are presumed to be neurotoxic and to cause DA cell death [20]. Mutation of the α-synuclein gene (*SNCA*) was found, in 1997, to cause a dominant fPD (PARK1) in which degenerating dopamine neurons contain both Lewy bodies containing α-synuclein and black pigment NM [9,10]. For these reasons, the α-synuclein protein has been extensively examined in relation to DA neuron death in sPD. However, a remaining question is that Lewy bodies are observed in dominant fPD such as PARK1 (*SNCA*), but not in recessive fPD such as PARK2 (*PARKIN*). (2) A black pigment NM, which is observed in the human SN, gradually increases during normal aging in healthy subjects [21]. NM is rich in the brain of humans, but the presence is also reported in the brain of monkeys, mice, rats, dogs, and horses [22,23]. Konstantin Tretiakoff [24], in 1919, reported that NM markedly decreased in the SN of PD brains. A decrease in NM in some nigrostriatal DA neurons in the SN pars compacta (SNpc), visible with the naked eye, are the main histopathological sign of PD. Different from Lewy bodies, NM is observed in sPD, dominant fPD, and recessive fPD. NM is also contained in norepinephrine (NE) neurons in the human locus coeruleus (LC), where NE neurons also degenerate in PD. In contrast to α-synuclein protein in Lewy bodies that has received great attention, the biosynthesis and pathophysiology of NM in PD remain less known. One reason is that elucidation of chemical structures of NM has been difficult owing to the small contents only in the postmortem human brains. However, the chemical properties and the biosynthesis pathway of NM has been elucidated in the last two decades based on the development of chemical micro-analysis of NM isolated from the SN of post-mortem human brains [25,26,27], and the pathophysiology of NM has also been gradually elucidated.

## 2. Biosynthesis of Neuromelanin (NM): Tyrosine Hydroxylase and Tyrosinase

**Figure 2:**
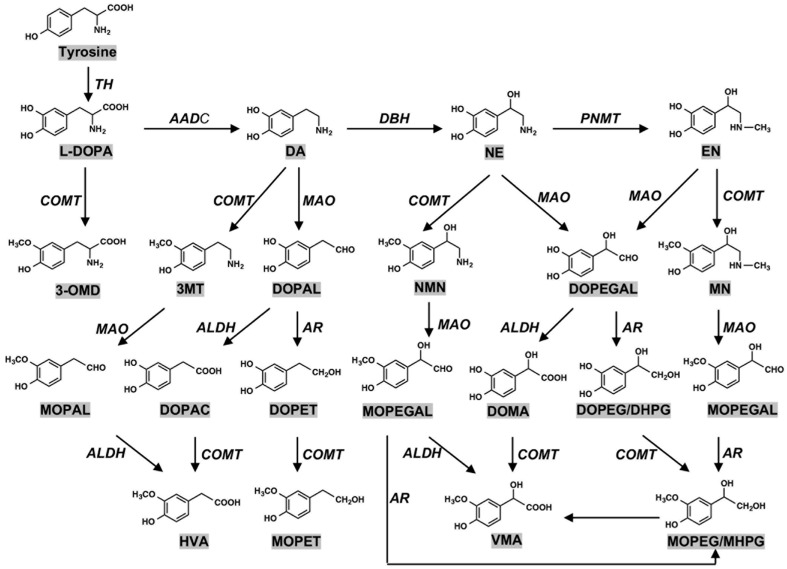
Metabolism of catecholamines. (DOPA) 3,4-dihydroxyphenylalanine; (DA) dopamine; (NE) norepinephrine; (EN) epinephrine; (3-OMD) 3-*O*-methyldopa; (3MT) 3-methoxytyramine; (DOPAL) 3,4-dihydroxyphenylacetaldehyde; (NMN) normetanephrine; (DOPEGAL) 3,4-dihydroxyphenylglycolaldehyde; (MN) metanephrine; (MOPAL) 3-methoxy-4-hydroxyphenylacetaldehyde; (DOPAC) 3,4-dihydroxyphenylacetic acid; (DOPET) 3,4-dihydroxyphenylethanol; (MOPEGAL) 3-methoxy-4-hydroxyphenylglycolaldehyde; (DOMA) 3,4-dihydroxymandelic acid; (DOPEG/DHPG) 3,4-dihydroxylphenylethyleneglycol/3,4-dihydroxyphenylglycol; HVA: homovanillic acid; MOPET: 3-methoxy-4-hydroxyphenylethanol; (VMA) vanillylmandelic acid; (MOPEG/MHPG) 3-methoxy-4-hydroxyphenylethyleneglycol/3-methoxy-4-hydroxyphenylgycol. (TH) tyrosine hydroxylase; (AADC) aromatic amino acid decarboxylase; (DBH) dopamine-β-hydroxylase; (PNMT) phenylethanolamine *N*-methyltransferase; (COMT) catechol-*O*-methyltransferase; (MAO) monoamine oxidase; (ALDH) aldehyde dehydrogenase; (AR) aldehyde reductase. Enzyme names are shown in italic for the sake of clarity. Adapted from [28] with minor modifications.

**Figure 3:**
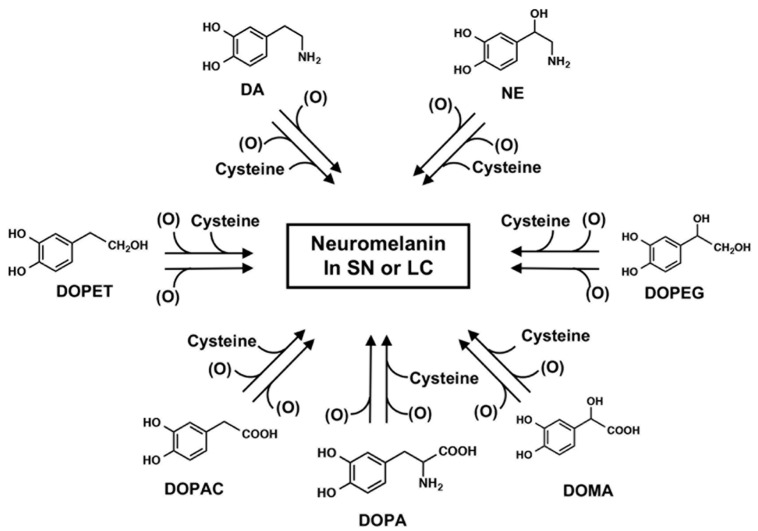
Synthesis of neuromelanin in SN or LC. Possible participation of various catecholamine metabolites known to be present in various regions of the brain that may be incorporated into NM in the substantia nigra (SN) or the locus coeruleus (LC). In addition to DA and NE and the corresponding Cys derivatives, these other metabolites are also thought to be incorporated into NM. (O) represents the oxidants. Taken from [29].

The pigmented NM in the human SN has been estimated to be derived from DA and cysteine at a molar ratio of 2:1 [27]. It has been reported that various catechol metabolites are incorporated into NM in the SN dopamine neurons and NE neurons in the LC, formed by oxidative deamination of catecholamines by monoamine oxidase (MAO) and following reduction and oxidation by aldehyde dehydrogenase (ALDH) and aldehyde reductase (AR): DOPA, 3,4-dihydroxyphenylacetic acid (DOPAC), and 3,4-dihydroxyphenylethanol (DOPET) as dopamine metabolites; 3,4-dihydroxymandelic acid (DOMA) and 3,4-dihydroxyphenylethylene glycol (DOPEG) as NE metabolites [27,28,29,30] (Figure 2). Based on these results, the pathway of NM biosynthesis via DA oxidation to DAquinone (DAQ) or via NE oxidation to NEquinone has been proposed to be similar to that of melanin biosynthesis involving the intrinsic pathway of DOPAquinone (DQ) in human skin and hair [31]. In addition, it has been suggested that various catecholic metabolites are incorporated into NM, including DOPA, DOPAC, DOMA, DOPET, and DOPEG, which are metabolites of DA and NE formed by the oxidative deamination by monoamine oxidase followed by oxidation/reduction [29] (Figure 3).

**Figure 4:**
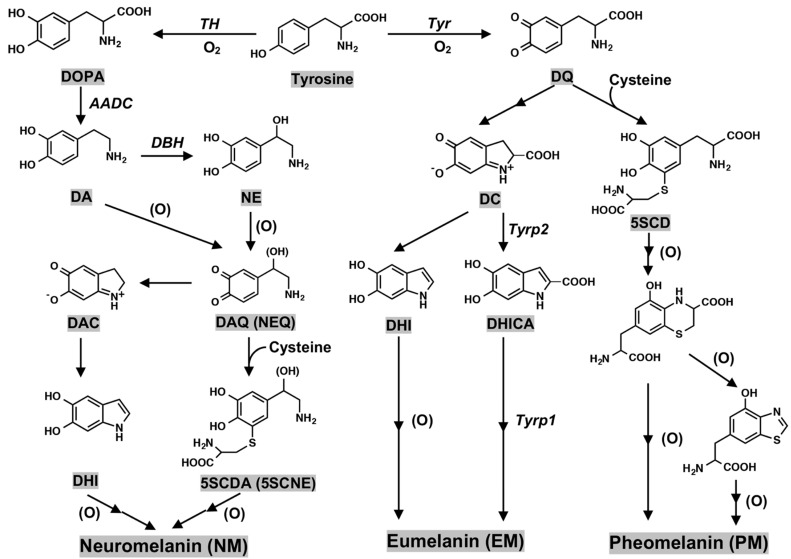
Biosynthesis pathway leading eumelanin, pheomelanin, and neuromelanin production. (DAQ) DAquinone; (NEQ) NEquinone; (DAC) DAchrome; (DHI) 5,6-dihydroxyindole; (5*S*CDA) 5-*S*-cysteinyldopamine; (5*S*CNE) 5-*S*-cysteinylnorepinephrine; (DQ) DOPAquinone; (DC) DOPAchrome; (DHICA) 5,6-dihydroxyindole-2-carboxylic acid; (5*S*CD) 5-*S*-cysteiyldopa; (Tyr) tyrosinase; (Tyrp2) tyrosinase-related protein 2; (Tyrp1) tyrosinase-related protein 1. Enzyme names are shown in italic for the sake of clarity. (O) represents the oxidants.

Peripheral melanin in human skin and hair is classified into two major pigments, i.e., black to brown pigments termed eumelanin (EM) and yellow to reddish brown pigments termed pheomelanin (PM); EM is synthesized in the absence of cysteine, and PM is synthesized in the presence of cysteine. NM synthesis in DA neurons is via dopaminequinone (DAQ)*,* whereas peripheral melanin synthesis in skin and hair is via DQ [29,30,31] (Figure 4). One more difference between the synthesis of NM and peripheral melanin is the presence in melanin synthesis of tyrosinase that is the rate-limiting enzyme in melanin synthesis in peripheral skin and hair [32,33,34,35], and the presence in NM synthesis of tyrosine hydroxylase (tyrosine-3-monooxygenase (TH)) that is the rate-limiting enzyme of catecholamine (DA, NE, and epinephrine (EN)) synthesis in DA and NE neurons. TH is an iron containing tetrahydrobiopterin (BH_4_)-dependent monooxygenase [36,37,38,39] (Figure 2).

Melanin in human skin and hair is synthesized by oxidation of L-tyrosine to DQ by copper-containing enzyme tyrosinase, in which DOPA is an auto-activator [32,33,34,35]. Since a shared genetic susceptibility between cutaneous malignant melanoma and PD has been suggested [40,41,42,43], an analysis of rare variants was carried out on cutaneous malignant melanoma genes in PD. The very rare tyrosinase gene variant, TYR p.V275F variant, is a pathogenic allele for recessive albinism, and was more common in PD cases than controls in three independent cohorts. Further studies in larger PD cohorts are needed to accurately determine the role of these genes/variants in disease pathogenesis [40,42]. The presence of NM was reported in the brains of 25 subjects with albinism, which are usually assumed to lack tyrosinase activity [44].

In the biosynthesis of human skin melanin under the absence of cysteine, DQ formed from tyrosine catalyzed by tyrosinase is further converted to dopachrome (DC) and then, via 5,6-dihydroxyindole (DHI) or via 5,6-dihydroxyindole-2-carboxylic acid (DHICA), the latter being catalyzed by tyrosinase-related protein 2 (Tyrp2, dopachrome tautomerase) [45,46]. Tyrosinase has an optimum pH of 7.4 and its activity is suppressed greatly at lower pH values [47]. The effects of pH (5.3–7.3) on the conversion of DC to DHI and DHICA and the subsequent oxidation of DHI and DHICA to form EM has been examined. Cu^2+^ can also catalyze this process [48]. Oxidative polymerization of DHI and DHICA in various ratios produces black to dark brown EM. Oxidative polymerization of DHI is catalyzed directly by tyrosinase or indirectly by DQ, while oxidation of DHICA appears to be catalyzed by tyrosinase-related protein 1 (Tyrp1; DHICA oxidase) at least in mice [49,50]. However, the human homolog TYRP1 may not act in the same way as in mice [51], and its precise enzymatic function in humans is not yet clear. In the presence of cysteine, DQ is converted to 5-*S*-cysteinyldopa (5SCD) and 2-*S*-cysteinyldopa (2SCD) as long as cysteine is present [52,53]. Oxidation of CD proceeds by redox exchange with DQ to form the quinone form. Cyclization and its rearrangement afford benzothiazine intermediates that are oxidized to form PM [54,55] (Figure 4).

In contrast to melanocytes in skin and hair, in the nigrostriatal DA neurons, the presence of tyrosinase for the oxidation of DA is still controversial [56,57,58,59,60,61]. In some studies, tyrosinase immunoreactivity was not detected in human SN neurons [58,61], while in other studies it was demonstrated that tyrosinase was expressed at low levels in human brain [57,59,60]. One study found that mRNA, protein, and enzyme activity of tyrosinase were all present but at barely detectable levels [60].

As described above, DA, which is the precursor of NM in the DA neurons, is synthesized from tyrosine by two enzymes: tyrosine is oxidized to L-DOPA by TH [36,38,39], and then L-DOPA is rapidly decarboxylated to DA by aromatic L-amino acid decarboxylase (AADC, also called DOPA decarboxylase (DDC)). Since both TH and AADC are cytosolic enzymes, DA formed in the cytoplasm, which is highly reactive and easily autooxidized, is rapidly transported into and stably stored in synaptic vesicles by vesicular monoamine transporter-2 (VMAT-2).

There are two hypotheses of synthesis of NM from DA in DA neurons. A common hypothesis is that DA synthesized from tyrosine by TH and AADC via DOPA is non-enzymatically converted by autoxidation probably with catalysis by iron or copper to eumelanic NM (euNM) and pheomelanic NM (pheoNM) in similar pathways as EM and PM synthesis catalyzed by tyrosinase [23,61,62]. It has been reported that in the presence of cysteine, DA was oxidized by Fe^2+^/Fe^3+^ or Mn^2+^ to form cysteinyldopamine (CDA) isomers and related metabolites [63]. Cu^2+^ can also oxidize DA [62,64]. In addition to these transition metals, catalyzed oxidations, reactive oxygen species (ROS) such as superoxide anion [65], hydroxyl radical [66], and hydrogen peroxide in the presence of peroxidase [67] are known to promote the oxidation of DOPA to produce 5-*S*-cysteinyldopa (5SCD) and 2-*S*-cysteinyldopa (2SCD). The other hypothesis assumes the presence of tyrosinase for pheoNM synthesis (as discussed above). In this connection, 5SCDA, the major isomer of CDA, was first detected in human brain in 1985 [68]. Then, it was detected in the homogenates of rat lung prepared in the presence of DA [69]. Elevated levels of 5SCDA were detected in guinea pig striatum and the levels increases with age [70]. This is an indication of DA oxidation taking place in SN, eventually leading to the formation of NM (pheoNM). L-DOPA has been reported to be a substrate of TH in the presence of SH compounds in in vitro activity assay. Theoretically, the oxidation of L-DOPA by TH may contribute to the formation of NM (pheoNM) [71].

In the absence of cysteine, DAQ is thought to be converted to dopaminechrome (DAC), and then via 5,6-dihydroxyindole (DHI) (Figure 4) to euNM. Interestingly, in *Drosophila,* an enzyme catalyzing the conversion of DAC to DHI was recently purified and identified [72]. Thus, it would be interesting whether this tautomerization activity is present in SN because DAC appears neurotoxic through binding to proteins [62,64]. In the presence of cysteine, DAQ is thought to be converted to 5SCDA and 2SCDA, and then converted to pheoNM [25,27,73]. In NE neurons in the LC, NE and cysteinyl-NE are thought to be incorporated into euNM and pheoNM, respectively [31].

The surface oxidation potential of human NM reveals a spherical architecture with a PM core and an EM surface [74]. This special arrangement of NM may protect neurotoxic pheoNM by surrounding protective euNM, as long as euNM is present enough. euNM is believed to act as a photoprotective antioxidant and pheoNM as a phototoxic prooxidant [74].

NM is composed together with complex aggregates of oxidized DA products, proteins, and lipids, which is most abundant in the SNpc [27,75,76]. NM pigments are contained within double membrane organelles along with lipid droplets and protein matrix [77]. The dominant lipid components are dolichol and dolichoic acid. A considerable number of glycolipids, glycerophospholipids, glycerolipids, and sphingolipids have also been found in NM [78,79]. These NM-containing organelles are a specific type of lysosomes derived from fusion with autophagic vacuoles [80]. The neuromelanin-containing organelle has a very slow turnover during the life of a neuron and represents an intracellular compartment of final destination for numerous molecules not degraded by other systems [81].

## 3. Neuromelanin (NM): The Cause of Parkinson’s Disease?

The pathophysiology of PD remains unknown. There are two hypotheses of cell death of DA neurons based on two histopathological hallmarks in PD, i.e., the α-synuclein hypothesis (an α-synucleinopathy) and the NM hypothesis (Figure 1). The α-synuclein hypothesis on the possible molecular mechanism of neuronal death of DA neurons in sPD may be summarized as follows: Mitochondrial oxidant stress by various exogeneous or endogenous factors may produce mitochondrial dysfunction, especially complex I deficiencies [82,83,84,85,86,87], oxidation of DA in cytoplasm [64,88,89], and formation of oxidized DA accumulation, especially toxic 3,4-dihydroxyphenylacetaldehyde (DOPAL), formation of toxic ROS, accumulation of cytotoxic fibrillar aggregates of α-synuclein oligomers, mitophagy/autophagy dysfunction, and neuroinflammation [90,91,92,93,94,95,96,97]. DOPAL is thought to accumulate in PD due to the low aldehyde dehydrogenase activity that oxidizes DOPAL to DOPAC in the SN in PD [98] and DOPAL generates potential reactive intermediates as causative agents for its neurotoxicity [99,100].

Since the 1990s, it was found that Lewy bodies mainly consisted of α-synuclein protein, and that the fibrillar oligomers produced by misfolding of the protein were neurotoxic and may be related to the cause of DA cell death [95,101,102]. In 1997, mutation of the α-synuclein gene (*SNCA*) was found to cause a familial PD (PARK1) [9,10]. Prion-like properties of α-synuclein were proposed by Braak (Braak hypothesis); α-synuclein produced in the intestine or olfactory bulb might spread via the vagus nerve or olfactory pathway to the midbrain and basal ganglia by cell-to-cell transfer [103,104,105]. α-Synuclein aggregates may spread from neuron to neuron, apparently transmitting the disease process through the brain. However, precisely how α-synuclein aggregates build-up and spread in this way is still unknown. Another question is that α-synuclein is not specific to PD, and also found in Lewy body disease (LBD) and multiple system atrophy (MSA) [106]. Aggregates of α-synuclein in distinct synucleinopathies, PD and MSA, have been proposed to represent different conformational strains of α-synuclein [107]. Even with these questions about the α-synuclein hypothesis, α-synuclein has been extensively examined in relation to DA neuron death in PD. The p62 protein normally assists in autophagy, a waste-management system that helps cells get rid of potentially harmful protein aggregates. In cell and animal models of PD, p62 is *S*-nitrosylated at abnormally high levels in affected neurons. This alteration of p62 inhibits autophagy, causing a build-up of α-synuclein aggregates, which in turn, leads to the secretion of segregates by affected neurons, and some of these aggregates are taken up by nearby neurons [108]. There are many references to support the cytotoxic effects of α-synuclein in vitro, especially in cell culture systems [10,20,95]. A downsized and optimized intracellular library-derived peptide prevents α-synuclein primary nucleation and toxicity without impacting upon lipid binding [109]. An animal model of PD with prodromal symptoms as in human PD has been reported [110]. The α-synuclein gene, *SNCA*, is a risk gene for sPD. A bacterial artificial chromosome transgenic mouse harboring *SNCA* and its gene expression regulating region in order to maintain the native expression pattern of α-synuclein showed prodromal symptoms in human PD such as RBD and anosmia without motor symptoms [110,111]. This mouse model is similar to human sPD and shows that α-synuclein alone can cause PD [110].

In the NM hypothesis, NM alone is related to DA neuron death. This review focuses on two independent ways of PD pathology, α-synuclein and NM. While both pathways may indeed lead to dopaminergic cell death, a decisive link between them is proposed “iron”, as pointed out recently by Riederer et al. [106,112,113]. The pathophysiology of NM decrease in the SN of DA neurons as a hallmark of PD remains unknown, especially in its relation to DA neuron death. In parallel with the elucidation of the chemistry and biosynthesis of NM in the DA neurons in the SN in PD, the physiological and pathological roles on NM have been studied since 2000s. NM in the SN increases gradually during aging in healthy subjects [21]. In contrast, NM decreases in PD. In PD, DA neurons containing NM in the human SN might preferentially degenerate, in parallel with the marked reduction in NM in the SN [114]. This fact, although controversial, suggests that NM is related to neurodegeneration and DA neuron death.

Alternatively, NM in DA neurons has generally been regarded as acting for neuroprotection, since NM inactivates toxic free radical species via its ability to chelate transition metals, especially iron. Iron also accumulates in DA neurons [112,115,116,117]. Iron is bound to NM in the ferrous (II) iron form, a redox-active form that is involved in a Fenton-like reaction to produce toxic free radical species. NM also eliminates various toxic substances in cytoplasm. Thus, NM may act for neuroprotection also in vivo. However, during the progress of PD, the release of toxic substances bound to NM owing to intracellular NM degradation may result in activation of microglia to release cytotoxic cytokines that produce neuroinflammation and neurodegeneration [78,118]. PD occurs spontaneously only in humans. To produce the PD phenotype in various models of PD in mammals such as in mice and rats that nearly lack NM in the brain, it is necessary to trigger the DA neurodegeneration by some toxic chemicals such as 1-phenyl-4-methyl-1,2,3,6-tetrahydropyridine (MPTP) that inhibits the mitochondrial complex I [97]. Vila’s group reported that NM accumulation in DA neurons during aging over a threshold causes DA neuron death and PD phenotype [119,120,121]. They created a rat model of human PD by overexpression of human NM in the right SNpc by stereotaxic injection of an adeno-associated viral (AAV) vector expressing human tyrosinase [119]. The rats showed age-dependent production of human-like NM within nigral DA neurons, up to levels in elderly humans. Intracellular NM aggregation above a specific threshold is associated with an age-dependent PD phenotype, including hypokinesia. Enhancing lysosomal proteostasis reduces intracellular NM and prevents neurodegeneration in tyrosinase-overexpressing rats. Intracellular NM levels may set the threshold for the initiation of PD. Furthermore, extracellular NM leaked from dead NM-containing DA neurons may activate microglia to produce neuroinflammation and to further promote DA cell death [122].

NM is a hot candidate to trigger PD and/or to lead to progressed degeneration of PD, however, there are unsolved problems arising from the treatment of PD with levodopa: (1) Post-mortem data have not shown an increase of NM in surviving NM containing dopaminergic neurons of the SN after levodopa long term treatment and (2) long term levodopa treatment has not demonstrated a significant increase in the progression of PD. The NM theory well fit to the phenotypes of human sPD. In addition, there is much evidence on the cytotoxicity of α-synuclein [10,19,123].

Recently, the role of NM in inducing α-synuclein expression and aggregation has been suggested as a mechanism for this pigment to modulate neuronal vulnerability in PD [124]. α-Synuclein reacts with tyrosinase, and the chemical modifications on the tyrosinase-treated α-synuclein strongly influence its aggregation properties and increase the toxicity, and α-synuclein may influence synthesis of NM [125,126]. Iron redox chemistry promotes the aggregation of α-synuclein, and protein-metal complex aggregates are directly involved in ROS production, exacerbating the oxidative damage [127]. Furthermore, DA neurons easily express MHC-I, and induction of MHC-I is promoted by activation of microglia either by α-synuclein or by NM, as well as by interferon gamma or high cytosolic DA and oxidative stress [128]. The activated microglia in PD brains express major histocompatibility complex class II (MHC-II) molecules. The number of MHC-II positive microglia in the SN and putamen increase as the neuronal degeneration of the SN proceeds [129].

An evolution theory has been proposed to explain human-specific PD based on the greater development of human cerebral cortex than that of basal ganglia [130,131,132]. Clinically, PD is a systemic disease, and it is difficult to explain the degenerative processes, especially in the autonomic nervous system, exclusively by NM theory, although there is accumulating evidence that the pathogenesis of PD is complex and involves energy metabolism disorders, oxidative stress, proteasomal abnormalities, α-synuclein accumulation, alterations of gut microbiota metabolites, and neuroinflammation [133,134]. In this context, the evolutional point of view on the NM system and α-synuclein system is also of interest.

## 4. Conclusions

Neuromelanin (NM) is thought to be synthesized by the following pathway: tyrosine →(TH)→ DOPA → (AADC)→ DA → (non-enzymatic oxidation or tyrosinase) → DAQ ----→ euNM/pheoNM. Finding neuromelanin-specific tyrosinase (activity) and DAC tautomerase (activity) remains for future study as an important problem in the pathophysiology of PD. NM is considered to act both for neuroprotection and for cell death of DA neurons depending on the intracellular levels of accumulation. The pathophysiology of NM in relation to α-synuclein is another important project for elucidating the cause of PD.

## Data Availability

Not applicable.

## Abbreviations

AADC	aromatic L-amino acid decarboxylase
AAV	adeno-associated viral
ALDH	aldehyde dehydrogenase
AR	aldehyde reductase
BH_4_	tetrahydrobiopterin
CD	cysteinyldopa
CDA	cysteinyldopamine
COMT	catechol-*O*-methyltransferase
DA	dopamine
DAC	dopaminechrome
DAQ	dopaminequinone
DBH	dopamine-β-hydroxylase
DC	dopachrome
DDC	dopa decarboxylase
DHI	5,6-dihydroxyindole
DHICA	5,6-dihydroxyindole-2-carboxylic acid
DQ	dopaquinone
DOMA	3,4-dihydroxymandelic acid
DOPA	3,4-dihydroxyphenylalanine
DOPAC DOPAL	3,4-dihydroxyphenylacetic acid 3,4-dihydroxyphenylacetaldehyde
DOPEG	3,4-dihydroxyphenylethylene glycol
DOPEGAL	3,4-dihydroxyphenylglycolaldehyde
DOPET	3,4-dihydroxyphenylethanol
EM	eumelanin
EN	epinephrine
euNM	eumelanic NM
HVA	homovanillic acid
LBD	Lewy body disease
LC	locus coeruleus
MAO	monoamine oxidase
MHPG	3-methoxy-4-hydroxyphenylgycol
MN	metanephrine
MOPAL	3-methoxy-4-hydroxyphenylacetaldehyde
MOPEG	3-methoxy-4-hydroxyphenylethyleneglycol
MOPET	3-methoxy-4-hydroxyphenylethanol
MOPEGAL	3-methoxy-4-hydroxyphenylglycolaldehyde
MPTP	1-phenyl-4-methyl-1,2,3,6-tetrahydropyridine
MSA	multiple system atrophy
NE	norepinephrine
NEQ	NEquinone
NM	neuromelanin
PD	Parkinson’s disease
fPD	familial Parkinson’s disease
sPD	sporadic Parkinson’s disease
pheoNM	pheomelanic NM
PNMT	phenylethanolamine *N*-methyltransferase
PM	pheomelanin
RBD	REM-sleep behavioral disorders
ROS	reactive oxygen species
5SCD	5-*S*-cysteinyldopa
2SCD	2-*S*-cysteinyldopa
SN	substantia nigra
SNCA	alpha-Synuclein gene
SNpc	substantia nigra pars compacta
TH	tyrosine hydroxylase
Tyr	tyrosinase
Tyrp1	tyrosinase-related protein 1
Tyrp2	tyrosinase-related protein 2
VMA	vanillylmandelic acid
VMAT-2	vesicular monoamine transporter-2
